# Hepadnavirus detected in bile and liver samples from domestic pigs of commercial abattoirs

**DOI:** 10.1186/s12866-014-0315-2

**Published:** 2014-12-11

**Authors:** Yasmine Rangel Vieira, Debora Regina Lopes dos Santos, Moyra Machado Portilho, Carlos Eduardo Pereira Velloso, Marcia Arissawa, Livia Melo Villar, Marcelo Alves Pinto, Vanessa Salete de Paula

**Affiliations:** Laboratório de Desenvolvimento Tecnológico em Virologia, Pavilhão Hélio e Peggy Pereira – 2° andar - sala B220, Instituto Oswaldo Cruz, FIOCRUZ, Av. Brasil, n° 4365, Manguinhos - RJ, Cep: 21045-900 Rio de Janeiro, RJ Brasil; Departamento de Mircobiologia Veterinária e Imunologia, Universidade Federal Rural do Rio de Janeiro, UFRRJ, Seropédica, RJ Brasil; Laboratório de Hepatites Virais, Instituto Oswaldo Cruz, FIOCRUZ, Rio de Janeiro, RJ Brasil; Laboratório de Tecnologia de Anticorpos Monoclonais, Bio-Manguinhos, FIOCRUZ, Rio de Janeiro, RJ Brasil

**Keywords:** Hepadnavirus, Brazil, Commercial swine, Bile, Liver, Genotyping, Immunofluorescence

## Abstract

**Background:**

Preliminary studies showed the prevalence of a virus similar to human hepatitis B virus (HBV-like) in swine from farms in China and the molecular evidence of Hepadnavirus infection in domestic pigs herds in Brazil. In this study, we genetically characterize the swine Hepadnavirus strains in swine from slaughterhouses located in certified abattoirs from Rio de Janeiro State, Brazil and evaluate its hepatotropic potential.

**Results:**

Bile and liver samples from swine were positive for partial genome amplification (ORF S and ORF C), direct sequencing and viral load quantification. Sequencing of the gene encoding the surface antigen allowed classification of Hepadnavirus into genotypes, similar to HBV genotype classification. Indirect immunofluorescence confirmed the presence of HBsAg antigen in liver tissue sections.

**Conclusions:**

So far our data suggest that commercial swine house an HBV-like virus and this relevant finding should be considered in studies on the origin and viral evolution.

## Background

Hepatitis B virus (HBV) is a dual polarity and partially double-stranded enveloped DNA virus of *Hepadnaviridae* family [1]. The agent can be transmitted by sexual, perinatal and percutaneous means [2], and is considered a major cause of acute and chronic liver disease, that may progress to cirrhosis and hepatocellular carcinoma [3]. Beyond the prototype member that infects humans and non-human primates (chimpanzees, gibbons, gorillas, orangutans and woolly monkeys), *Hepadnaviridae* family also houses HBV-related viruses circulating in mammalian hosts, like woodchuck (WHV) and squirrels (GSHV/ASHV), and avian hosts, like ducks (DHBV), geese (GHBV), herons (HHBV), and storks (STHBV) [4].

A virus similar to HBV has been diagnosed by serology (HBsAg, anti-HBs, anti-HBc) in swine herds [5] and chickens flocks [6] from China, and in domestic pig herds in Brazil [7]. Moreover, positive molecular diagnosis was demonstrated for the first time in swine from Brazil [7] and in chickens flocks from China [6]. Similarity with human HBV (90.8-96.3%) was confirmed for swine strains by phylogenetic analysis and by cross reactivity in non-host specific commercial serological assays. However, the partial nucleotide sequencing (360 bp) was equivalent to about 11.2% of full-length HBV genome. And even for chickens, despite the high percentage of similarity with HBV (92.2-97.9%), the short length of amplified product in both cases limits a conclusion.

Therefore, we performed the current study to improve the molecular characterization of Hepadnavirus circulating in swine from abattoirs in Brazil, revealing if there is evidence that pigs destined for human consumption might act as a potential new reservoir or host for a virus HBV-similar.

## Methods

This study was approved by the Institutional Committee for Ethics in the Use of Research Animals (CEUA-Fiocruz: PO 0132/01). On December 2008, a total of 36 bile and liver samples were collected from domestic pigs *Sus scrofa* (aged > 5 months), breed Large white, from three slaughterhouses located in Petrópolis (SPET), Itaocara (SITC) and Itaperuna (SITP) (North and Hill region of Rio de Janeiro State, Brazil). Commercial establishments are submitted to controlled inspection by an official agency of Rio de Janeiro State. All animals from the three abattoirs were classified healthy and approved for slaughter and further commercialization according to inspect evaluation criteria. Swine from each abattoir were acquired from distinct pig farms suppliers. The samples were collected during the evisceration process under sanitary requisites determined by regulations of Animal Sanitary Protection Agency in Rio de Janeiro State (ASPA).

Five milliliters of bile were collected by vaccum-punction through the gallbladder wall with sterile syringe; and 500 mg of liver samples were collected with Medblade® bisturi blades. All samples were stored in Nalgene® cryogenic vials and immediately frozen in dry ice. At the laboratory, bile samples were stored at -80°C and liver samples in liquid nitrogen until analysis. Viral DNA was extracted from bile and liver samples using DNA Purification Kit (QIAamp DNA Mini Kit, Qiagen®) according to Moricz et al, 2010 [8] and was concentrated to a final volume of 25 uL. Extracted DNA was analyzed by semi-nested PCR (PS1-S2 and PS1-SR) specific for open reading frame (ORF S) of HBV - as previously established for the first round of amplification [9], and for the second round [10] - and by PCR specific for core gene of HBV – as previously established [11]. Direct sequencing of amplicons was performed to identify the sequence amplified.

After molecular diagnosis, in order to quantify the viral load DNA samples were evaluated in duplicate by Real Time PCR using TaqMan® method. The Real Time PCR assay was performed for pre-S2/S region as previously established [12], using the following primers pair and fluorescent probe: forward primer (5’-GAATCCTCACAATACCGCAGAGT-3’), reverse primer (5’-GCCAAGACACACGGGTGAT-3’), and probe (5’-FAM-AAGTCCACCACGAGTCTAG-NFQ/MGB-3’) (Life Technologies®, Applied Biosystems). HBV plasmid serially diluted from 5 × 10^7^ to 5 × 10^1^ copies/ μL was used as HBV standard PCR template. The amplification was analyzed using the software Applied Biosystems 7500.

Indirect immunofluorescence assay was also performed in samples of liver tissue cryosectioned at 4 μm. Liver biopsies were stained with anti-HBs mouse monoclonal primary antibody specific for human HBsAg (19-CC6/CG2 Lot 100830S019, Biomanguinhos, Fiocruz, Rio de Janeiro, Brazil) at the dilution 1:50 overnight at 4°C, followed by Alexa Fluor 488 conjugated goat anti-mouse secondary antibodies (Molecular Probes®) at the dilution 1:400 for 1 h at 37°C. Liver sections were counterstained with Evans Blue (1: 20,000) and DAPI (Molecular Probes®). Images of the green fluorescent HBsAg-positive liver cells were observed and photographed using a Confocal Scanning Laser Microscope equipped with camera (Nikon® Instruments, Model C2, Inc., New York, USA).

## Results

**Figure 1:**
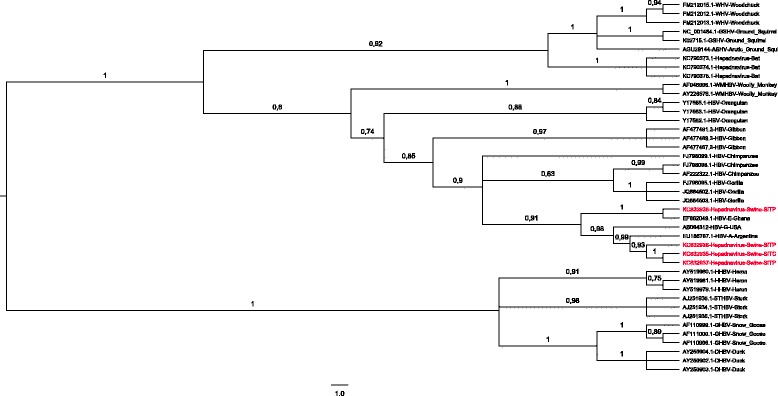
**Phylogenetic analysis of swine Hepadnavirus strains and** ***Hepadnaviridae*** **family.** The Bayesian phylogenetic tree was constructed by using partial nucleotide sequence of open reading frame S (566 nt) of HBV and related viruses. For each sequence used, the GenBank accession number and country of isolation are shown. Multiple nucleotide sequence alignment was analyzed by using the Markov Chain Monte Carlo method implemented in the program MrBayes version 3.1.2 under GTR + G nucleotide substitution model, selected using the jModeltest program. The tree was rooted at midpoint. Posterior probabilities are shown at the branch label. Newly described swine hepadnavirus sequences are indicated. Scale bar indicates evolutionary distance.

Hepadnavirus-DNA was detected in bile and liver samples from about 11.11% pigs (4/36). All samples from Petrópolis (0/9) were tested negative. Hepadnavirus-DNA was detected in both samples types from 1 animal in Itaocara (1/10) and from 3 animals in Itaperuna (3/17). These samples showed a positive result for the semi-nested PCR specific for HBV ORF S (1,100 bp) [GenBank:KC832935, GenBank:KC832936, GenBank:KC832937, GenBank:KC832938], and 3 of them also for the PCR specific for core gene (431 bp) [GenBank:KF859967, GenBank:KF859968, GenBank:KF859969]. The sequences found in bile and liver of the same animal matched. Phylogenetic reconstruction using partial nucleotide sequences of ORF S (566 nt) showed a close relationship of Hepadnavirus strains from pigs and Hepadnavirus nucleotide sequences from non-human primates (from 84.8 to 96.1%), bats (from 68.9 to 75.6%), rodents (from 70.5 to 71.8%) and birds (from 45.8 to 48.6%) (Figure 1, Table 1).

**Table 1:** **Nucleotide identity between Hepadnavirus strains from pigs and other species**

**Species**	**Accession Number GenBank**	**Swine Hepadnavirus**			
		**KC832935**	**KC832936**	**KC832937**	**KC832938**
**Chimpanzee** (HBV)	FJ798099.1	0.950	0.950	0.951	0.961
	AF222322.1	0.937	0.937	0.939	0.948
**Gibbon** (HBV)	AF477494.2	0.939	0.939	0.940	0.953
	AF274499.2	0.918	0.918	0.920	0.933
**Orangutan** (HBV)	Y17562.1	0.916	0.918	0.918	0.929
	Y17559.1	0.911	0.913	0.913	0.926
**Gorilla** (HBV)	AJ131567.1	0.946	0.946	0.948	0.951
	JQ664502.1	0.942	0.942	0.944	0.948
**Woolly Monkey** (WMHBV)	AF046996.1	0.850	0.848	0.851	0.848
	AY226578.1	0.850	0.848	0.851	0.848
**Bat** (Hepadnavirus)	KC790378.1	0.689	0.689	0.691	0.695
	KC790376.1	0.755	0.753	0.756	0.755
**Woodchuck** (WHV)	FM212013.1	0.707	0.705	0.709	0.707
	FM212009.1	0.716	0.714	0.718	0.714
**Squirrel** (ASHV/GSHV)	AGU29144	0.709	0.709	0.710	0.712
	K02715.1	0.709	0.709	0.710	0.716
**Heron** (HHBV)	AY552597.1	0.474	0.477	0.476	0.481
	AY552595.1	0.483	0.484	0.484	0.486
**Stork** (STHBV)	AJ251937.1	0.464	0.466	0.466	0.471
	AJ251935.1	0.464	0.466	0.466	0.468
**Snow Goose** (GHBV)	AF110999.1	0.458	0.461	0.460	0.461
	AF110997.1	0.461	0.465	0.463	0.465
**Duck** (DHBV)	AY494851.1	0.467	0.470	0.468	0.461
	M21953.1	0.473	0.476	0.475	0.476

Legend: The nucleotide identity matrix was constructed by using partial nucleotide sequence of open reading frame S (566 nt) of HBV and related viruses. For each sequence used, the GenBank accession number and specie infected are shown. Newly described swine hepadnaviruses sequences are indicated.

**Figure 2:**
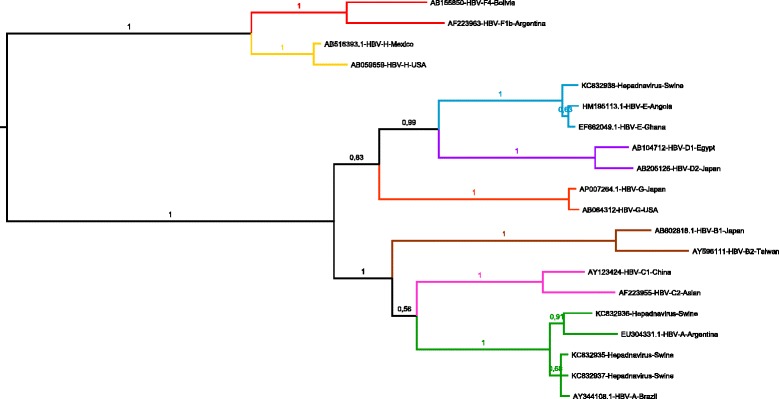
**Phylogenetic analysis of swine Hepadnavirus strains and HBV from human samples.** The Bayesian phylogenetic tree was constructed by using partial nucleotide sequence of open reading frame S (972 nt) of HBV. For each sequence used, the GenBank accession number and country of isolation are shown. Multiple nucleotide sequence alignment was analyzed by using the Markov Chain Monte Carlo method implemented in the program MrBayes version 3.1.2 under GTR + G nucleotide substitution model, selected using the jModeltest program. The tree was rooted at midpoint. Posterior probabilities are shown at the branch label. Newly described swine hepadnavirus sequences are indicated. Scale bar indicates evolutionary distance.

Genetic distances calculated with nucleotide sequences of ORF S (972 nt) showed filogenetic distance between the virus detected in Brazilian domestic pigs and human samples from significant alignment listed by NCBI Blast [GenBank:JF784228, GenBank:JF784230, GenBank:JF784231, GenBank:JF784234, GenBank:EF662027-49, GenBank:DQ060826, GenBank:DQ060829] ranging from 98.9-99.7%. These data also revealed that distinct strains might be responsible for the infection in domestic pigs (Figure 2). Comparing to human HBV strains previously genotyped, swine samples were assigned to two different genotypes groups. Three of them were close to samples from genotype A, the most prevalent genotype in Brazil [13]. And, another was similar to samples that belong to genotype E, which is common in western Africa [14]. Genotype A was found circulating in Itaocara and Itaperuna, while genotype E was found exclusively in Itaperuna.

Concerning viral load quantification, a linear relationship was obtained between the cycle threshold (Ct) values and the log_10_ concentration of the HBV DNA. The regression analysis yielded a correlation coefficient of 0.99. All four bile samples could be quantified. The viral loads ranged from 0.8 × 10^3^ to 1 × 10^5^ copies/mL.

**Figure 3:**
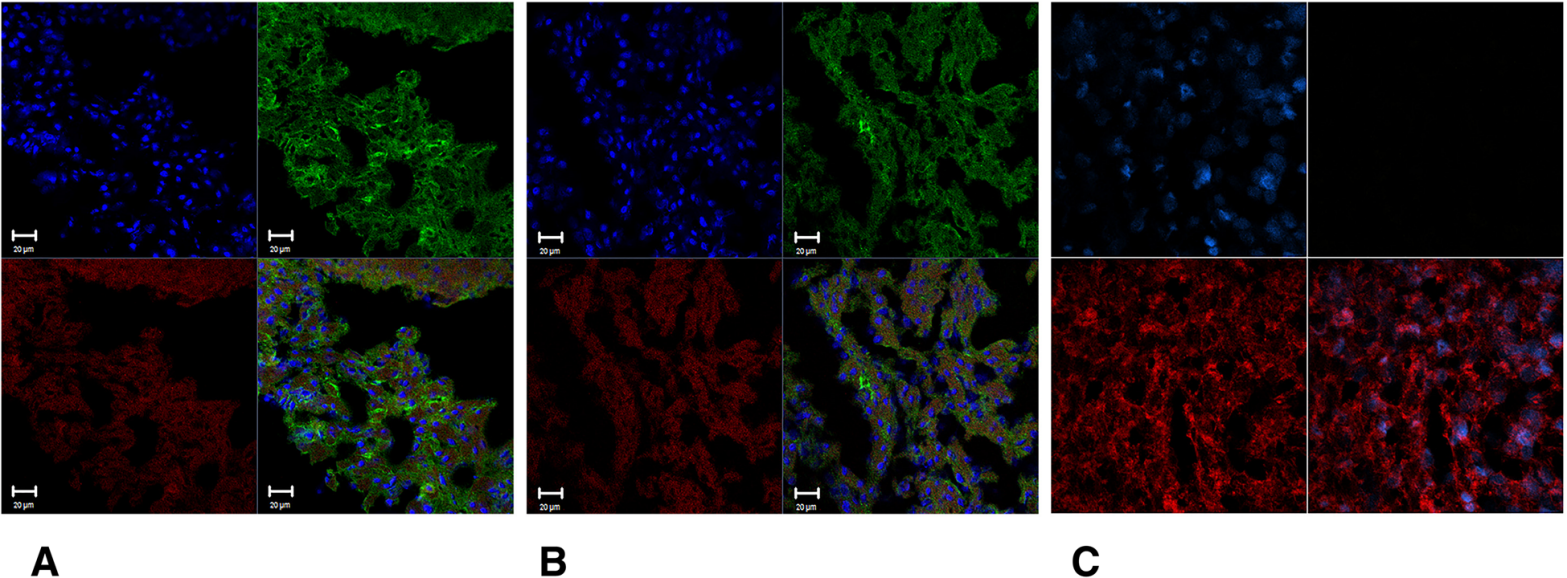
**Detection of hepatitis B virus surface antigen in liver of swine by immunofluorescence staining.** Different frozen sections of HBsAg-positive cells **(A **and **B)** from liver of pigs stained in green using Alexa Fluor 488-conjugated anti-mouse IgG as secondary antibody. Evans Blue (red) and DAPI (blue) counterstained all liver sections. Images were examined under a confocal immunofluorescent microscope (Original magnifications x40). Liver sections from negative Hepadnavirus-DNA animals were used as negative controls **(C)**.

Further, we investigated viral hepatotropism by indirect immunofluorescence analysis using monoclonal anti-HBs antibody in liver biopsies from *Hepdnavirus*-DNA positive animals, and compared the results to the control animals. HBsAg was observed in cell membrane and cytoplasm of sinusoidal lining cells in liver parenchyma and in hepatocytes (Figure 3). No staining was observed for controls. Molecular data and antigenic detection in situ confirm that swine were infected by a Hepadnavirus with hepatotropic behavior, similar to human HBV.

## Discussion

Our challenge in this study was to refine the molecular characterization of Hepadnavirus circulating in swine from Brazil, disclosing how close porcine strains are to HBV and to HBV-like viruses sequences described for different hosts, including human. Data presented here showed different strategies for viral detection, including the molecular characterization of complete ORF C and ORF S in bile and liver samples, the genotype classification based on the genotypic classification of HBV, the viral load quantification and antigenic detection in hepatic parenchyma.

This is the first record of molecular characterization of swine Hepadnavirus from bile and liver samples. There are no previous data available in the literature concerning genotypic classification based on complete sequencing of pre-S/S region and viral load evaluation in swine from slaughterhouses.

According to results described previously for Brazilian domestic swine [7], the similarity between pigs and human Hepadnavirus strains was supposed through superficial and preliminary estimates, and cross reactivity in non-host specific commercial serological assays. In this study, molecular results from a larger and more representative fragment of the genome allowed us to calculate an approximate identity not only with HBV from human host, as well as other viruses that comprise the *Hepdnaviridae* family: 98.9-99.7% to human HBV, 93.7-96.1% to Chimpanzee HBV, 91.8-95.3% to Gibbon HBV, 94.2-95.1% to Gorilla HBV, 91.1-92.9% to Orangutan HBV, 84.8-85.1% to Woolly Monkey WMHBV, 68.9%-75.6% to Bat Hepadnavirus, 70.5-71.8% to Woodchuck WHV, 70.9-71.6% to Arctic/ Ground Squirrels ASHV/ GSHV, 47.4-48.6% to Heron HHBV, 46.4-47.1% to Stork STHBV, 45.8-46.5% to Snow Goose GHBV, 46.1-47.6% to Duck DHBV.

Genetic distances data are relevant and need to be better evaluated once despite the diversity of hosts, no reservoirs are described for HBV so far [15]. At the same time, as commercial swine are slaughtered at a young age (up to 22 weeks old), the identification of any clinical signs of disease may be hindered notably considering that health inspections are based only in visible lesions present and post-mortem gross macroscopic examinations in animal from abattoirs. No molecular tests are executed. Due to these factors, a follow-up study for longer periods should be considered.

It is noteworthy that investigation of the population of animals from three different commercial herds was performed after a single collection, and 11.11% of swine showed positive molecular diagnosis, in which hepatotropism was also confirmed. These positive animals were distributed in two slaughterhouses which do not share the same pig farm supplier.

The possibility of an eventual vertical transmission can not be ruled out for positive animals from the same slaughterhouse since they came from the same herd. However, as there are distinct breeding sows in a single pig farm, animals destined to the same abattoir may be born from different sows. To consider the risk of vertical transmission, pigs should also share the same genotype in addition to sharing the same geographical origin.

In this study, three positive animals came from Itaperuna. One animal was assigned to genotype E, and two animals shared genotype A. Due to the circulation of both genotypes in swine from the same abattoir, at least two different breeding sows were involved in its respective pig farm supplier. Even though, the risk of vertical transmission for two animals of genotype A cannot be confirmed.

Since the role of bats as reservoirs of zoonotic viruses is now being investigated, including for bat hepadnaviruses [15], further studies are also necessary to extrapolate this reasoning to the pigs analogously. Whether there is a zoonotic ability of swine Hepadnavirus strains and if this virus is able to induce cross-infection in other species are remaining issues. Considering the main routes of HBV transmission, and since personal protective equipment is not often used in slaughterhouses, novel studies are necessary to assess a potential occupational risk of infection by Hepadnaviruses during the management of pigs and their derivatives.

## Conclusions

The present study demonstrated the circulation of an emerging virus in swine, similar to Hepadnavirus that causes human infection. Further studies are necessary to disclose the actual role of swine in the viral cycle.

## Abbreviations

HBV: Human hepatitis B virus
ORF S: Open reading frame S
ORF C: Open reading frame C
WHV: HBV-related virus of woodchuck
GSHV: HBV-related virus of ground squirrels
ASHV: HBV-related virus of arctic squirrels
DHBV: HBV-related virus of ducks
GHBV: HBV-related virus of geese
HHBV: HBV-related virus of herons
STHBV: HBV-related virus of storks
HBsAg: Surface antigen of HBV
anti-HBs: HBV surface antibody
anti-HBc: Antibody to HBV core antigen
bp: Base pair
CEUA: Committee for Ethics in the Use of Research Animals
ASPA: Animal Sanitary Protection Agency in Rio de Janeiro State
nt: Nucleotide
